# Evolution of Scientific Production on Phlebitis Secondary to Vascular Access: A 71-Year Bibliometric Analysis

**DOI:** 10.3390/nursrep13040135

**Published:** 2023-11-13

**Authors:** Alba Torné-Ruiz, Judith García-Expósito, Aida Bonet, Olga Masot, Judith Roca, Laia Selva-Pareja

**Affiliations:** 1 Department of Nursing and Physiotherapy, University of Lleida, 25199 Lleida, Spain; alba.torne@udl.cat (A.T.-R.); aida.bonet@udl.cat (A.B.); olga.masot@udl.cat (O.M.); laia.selva@udl.cat (L.S.-P.); 2 Hospital Fundació Althaia, Xarxa Assistencial Universitària de Manresa, 08243 Manresa, Spain; 3 Group Preving (Vitaly), 03003 Alicante, Spain; 4 Health Care Research Group (GRECS), Biomedical Research Institute of Lleida, 25198 Lleida, Spain; 5 Health Education, Nursing, Sustainability and Innovation Research Group (GREISI), 25199 Lleida, Spain

**Keywords:** bibliometrics, catheter, phlebitis, thrombophlebitis, vascular access devices

## Abstract

Phlebitis secondary to vascular access is one of the most frequent complications in hospital care. This study aims to evaluate the scientific activity related to this complication through a bibliometric analysis. The search was performed on a single day, 23 January 2023, to ensure the inclusion of all articles and to avoid bias caused by the daily updates of the open access database. The data were recovered from Web of Science. The sample comprised a total of 1596 publications that met the inclusion criteria. The United States was the country with the largest number of publications, citations, and international cooperation with respect to phlebitis and vascular access. The most important author was Rickard CM. Of all the publications selected, a total of 1586 (99.37%) were original articles. The highest number of articles on the subject was recorded in 2021, and the most common research areas were General Internal Medicine and Nursing. The analysis of the clusters (KeyWords Plus and Author keywords) and co-occurrences enabled identification of areas of interest and their possible development. These areas included the prevention, risk, and associated complications of catheter-associated phlebitis. Other aspects that are a priori relevant, such as assessment and treatment, were found to be little investigated. While research on this subject is increasing internationally, more collaborations are still required between researchers, as well as new approaches related to the management of catheter-associated phlebitis. The dimensions that should continue to be considered in new research, according to the findings of this review, are instruments for phlebitis assessment and their validation, and the treatments to follow in the case of established phlebitis. For this reason, the bibliometric information presented is key for new or consolidated researchers in the field, especially because of its practical and clinical implications for patient safety.

## 1. Introduction

One of the most common procedures performed during hospital care is the placement of a venous catheter [1]. It is estimated that 70% of patients have a peripheral venous catheter (PVC) during their hospital stay [2]. Despite the multiple advantages of its use, it is associated with some complications, such as phlebitis, infiltration, and to a lesser degree, occlusion and catheter dislodgement [3,4]. It can also cause patients serious complications that require the use of antibiotics or even surgical intervention, causing an increase in the length of the hospital stay [5]. 

Phlebitis is one of the most frequent epidemiological complications. It is an inflammation of the tunica intima of a superficial vein caused by mechanical, chemical, or bacterial sources [6,7,8]. However, it should be noted that phlebitis associated with vascular access has not yet been clearly defined [9]. This has been observed in different studies, in which phlebitis has been associated with different signs and symptoms [10,11]. In addition, a wide variety of instruments have been employed to assess phlebitis [12,13]. 

There is no consensus on the optimal management of PVC-associated phlebitis at the clinical level, although several treatments have been proposed in the literature, including topical and systemic types [3,9,14]. The guidelines that have been proposed are non-specific and do not discuss the pharmacological or therapeutic interventions that should be carried out to minimize the signs and symptoms of patients suffering from phlebitis. The analysis of treatments and their possible effects on the symptoms associated with phlebitis due to venous catheter placement is fundamental, given the high incidence in the clinical context, the need to improve nursing knowledge with respect to this practice, and the associated risks [15]. Nursing personnel are the main individuals responsible for catheter care, and it is very important to strengthen their basic knowledge about hand hygiene and aseptic techniques, the selection of catheters and locations, site bandaging regimes, preparation of the skin, replacement of administration equipment, and systems of intravascular catheters without needles [3].

However, little is known about the scientific production in this regard or the trend of future research at the international level. Only one national study considers these questions [16]. The need to qualify, quantify, and understand the research conducted in this area of health can be met through a bibliometric analysis. The search question formulated for the present study is: “How has the scientific literature on phlebitis secondary to vascular access evolved over time and what are the main elements that characterize it, including influential authors, publication trends, geographic areas and publication languages?”.

This type of analysis is a recent phenomenon, with an increasing trend in nursing research [17,18]. The field of bibliometrics is closely related with scientometrics, and its objective is to identify the corpus of the literature, as well as its quantitative and statistical analysis. These analyses are necessary for assessing the scientific and clinical activity in a field, as they provide essential information on the current state of progress in specific branches in particular [19,20,21]. Thus, the impact of the scientific literature can be analysed and interpreted for its use in research [19].

This study aims to obtain an overview and evaluate the evolution and development of the scientific literature available on phlebitis secondary to vascular access. The secondary objectives include: (1) analysis of the contributions as a function of publication year, of influential authors in terms of citation scores, and of collaborations with other authors; (2) determination of the publication trend in journals and research areas; (3) identification of the geographical area and publication language of the documents; and (4) identification of keywords and their co-occurrence through visual networks of terms/words.

## 2. Materials and Methods

A descriptive bibliometric analysis was performed. The methodological steps used in a study carried out by Selva et al. [22] were followed, based on the three phases described by Fauzi [23] of data collection, screening, and analysis.

### 2.1. Stage 1: Data Collection

#### 2.1.1. Literature Research

An extensive systematic search was performed of the literature. The source of data for the present study was the Web of Science (WOS), due to its interconnections and citations in various areas of research. This database also contains the oldest and most complete records of citation indices, and includes a useful analysis tool [19]. The search was performed in the WOSCC database, which allowed for a more precise and specific analysis of the publications, the authors, the citations, and the keywords [24]. To be able to synthesize all the information, different software programs were utilized, such as Bibliometrix 3.1.4 for RStudio (Department of Economics and Statistics, University of Naples Federico II, Napoli, Italy) and VOSviewer (Centre for Science and Technology Studies, Leiden University, Leiden, The Netherlands). To create a representative corpus of documents for the study, a search strategy was created that included related keywords, which were obtained from the MESH terms from PubMed: TS = (“phlebitis or thrombophlebitis” and “catheter”).

#### 2.1.2. Identifying Relevant Studies

To identify relevant articles and include them in our study, some inclusion criteria were determined: (a) articles that were published on the subject; and (b) original articles in any language. The exclusion criteria were: (a) articles that were considered non-original (reviews and other documents such as books, letters to the editor, editorials, and notes, among others).

The search was performed on a single day, 23 January 2023, to ensure the inclusion of all the articles, to avoid bias caused by the daily updating of the databases, and to be able to present a static view of the field at that point in time.

### 2.2. Stage 2: Screening

#### 2.2.1. Eligibility Criteria

To ensure the quality of the articles included in the study, a filter based on ‘document type’ was applied. Additionally, when reviewing the titles and abstracts of the articles, the ‘document type’ criterion was again used to determine which articles met the inclusion requirements and which should be excluded. 

#### 2.2.2. Study Selection and Data Collection

A bibliometric analysis with relevant information was extracted, such as: year of publication, number of references, authors, publication journals, areas of interest and research, countries, languages, and keywords utilized. To select the studies, two authors (JG and AT) assessed the titles independently. Any disagreements that emerged were resolved by consensus with a third author (JR). To select the articles, the methodological approach known as “Preferred Reporting Items for Systematic Reviews and Meta-Analysis” (PRISMA) was used.

### 2.3. Stage 3: Analysing the Data

#### 2.3.1. Performance Analysis

First, a descriptive bibliometric analysis was carried out with the WOS tool. Information was extracted including publication year and record count, Web of Science Index, author information and affiliations, document types, and index H. Likewise, information was consulted from all the journals in which an article related to the topic had been published.

#### 2.3.2. Science Mapping

For the data analysis, the open-source software RStudio was utilized, with the R package Bibliometrix 3.1.4, which provides a set of tools for quantitative research in bibliometrics and scientometrics [25,26]. Additionally, to show the data through time, the data downloaded from the WOS were imported into VOSviewer. This software can be used for constructing and visualizing co-occurrence networks of important terms extracted from both the titles and the abstracts of the scientific literature through the representation of keywords with different colours and circles of different sizes [27]. The hotspots or critical points of the clusters were defined as keywords from recent popular scientific fields, and their frequency of appearance was calculated [28].

For these analyses, different variables were used: citations, affiliations, countries, publication language, and keywords.

## 3. Results

**Figure 1:**
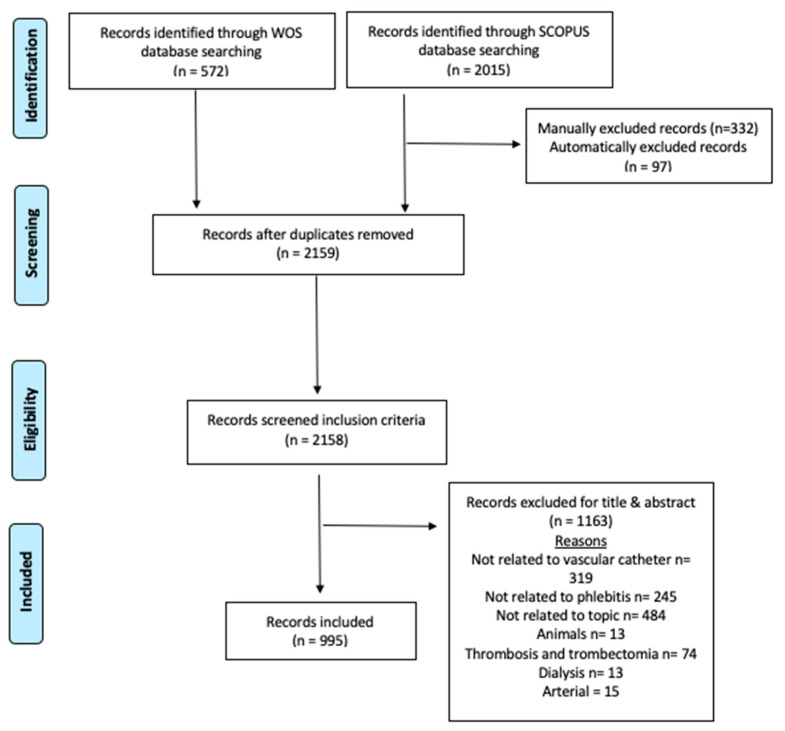
Flow diagram adapted from the Preferred Reporting Items for Systematic Reviews and Meta-Analyses (PRISMA) guidelines.

The search performed in the WOSCC database provided a total of 890 articles, which was reduced to 586 articles after application of the inclusion and exclusion criteria and their evaluation according to their title and abstract. The process is shown graphically in Figure 1 with a PRISMA flow diagram.

### 3.1. Year of Publication, Number of Citations, and Authors

The first publication on the subject was written in 1951, and the number of related publications was scarce until 1991, when production increased. In 2015, the number of publications strongly increased with respect to the previous years. The highest number of articles (n = 51) was found in 2021.

**Figure 2:**
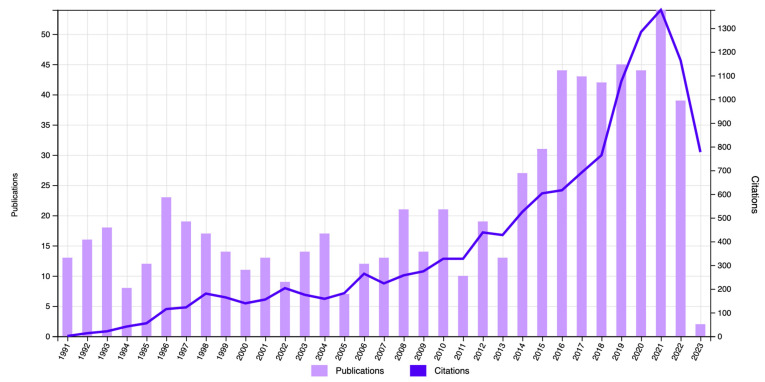
Number of publications and citations according to year in the WOSCC database (1991–2023).

As for the number of citations included in the bibliometric study, the articles were cited a total of 5303 times (without self-citations) throughout the years, with the most citations (n = 1198) found in 2021 (Figure 2). The analysis of citations excluding self-citations ensures a more objective perspective that better reflects the scientific production.

**Figure 3:**
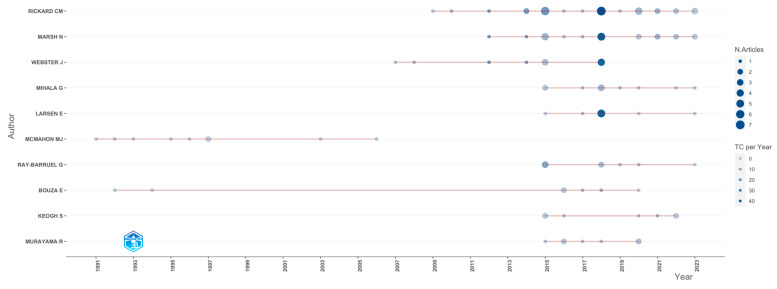
Author production over time.

The author who received the most citations (n = 4685) was Rickard CM. The same author was also the most productive (34 articles), followed by Marsh N. with 22 articles, and Mihala G. with 17 articles (Figure 3).

**Figure 4:**
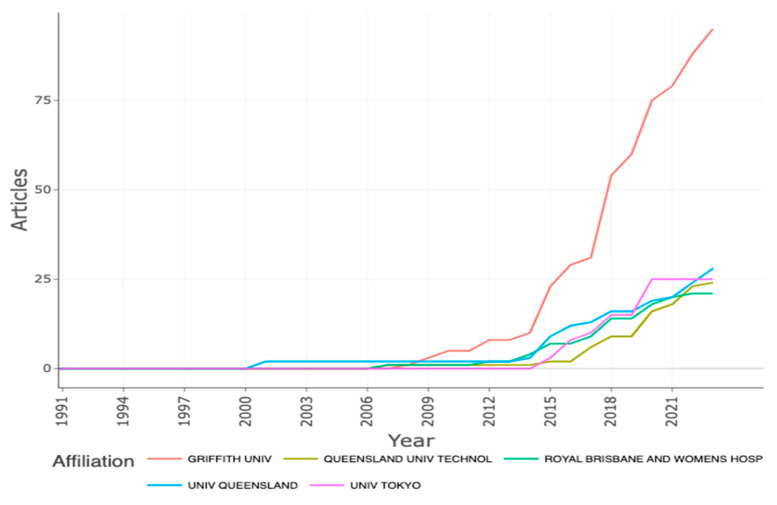
Affiliation production over time.

It is important to mention that these three authors had the same affiliation, the Menzies Health Institute, Queensland. This institution was in third place with respect to production, with a total of 27 records, and was the first and only to publish about the subject from 2001 to 2006. Ahead of this institution was the Griffith University (n= 37) and the Royal Brisbane Women’s Hospital (n = 32) (Figure 4).

### 3.2. Journals, Areas of Research

A total of 318 journals disseminated articles on phlebitis and vascular catheter access, but those with the highest number of publications were: *Journal of Vascular Access* (n = 27, 4.61%), *Journal of Infusion Nursing* (n = 14, 2.39%), *Journal of Clinical Nursing* (n = 13, 2.22%), *Journal of Parenteral and Enteral Nutrition* (n = 13, 2.22%), and *Clinical Nutrition* (n = 8, 1.36%). According to the Journal Impact Factor (JIF), the impact factor of the five journals with the most publications ranged between 0.789 and 7.643, and encompassed three different areas of knowledge, with two of them being Q1 journals (Table 1).

As for the areas of research, the following can be highlighted: Nursing with 116 records, General Internal Medicine with 101, and Infectious Diseases with 61.

### 3.3. Countries and Publication Language

**Figure 5:**
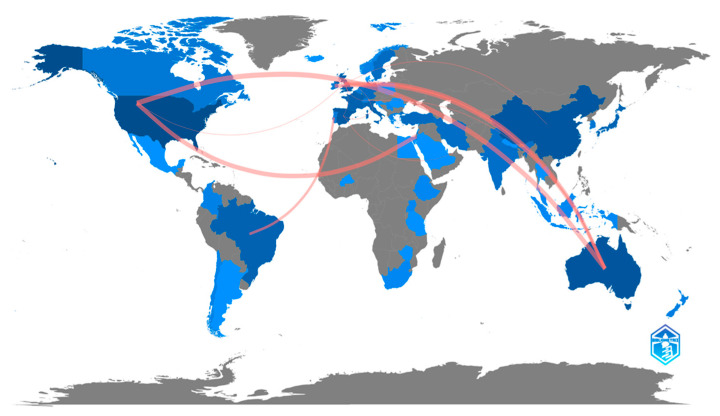
Countries with the greatest scientific production on phlebitis and vascular access catheters, and collaborations established. The intensity of the blue color indicates a greater contribution to scientific production. The grey color indicates no scientific production about the subject. The red lines indicate collaboration between countries.

Among the countries with the highest number of publications, we found: the USA (n = 116, 23.21%), Australia (n = 64, 10.92%), China (n = 48, 8.19%), and Spain (n = 47, 8.02%). As for collaborations between different countries, the countries with the most collaborations were Australia–USA (6), USA–Israel (5), Australia–United Kingdom (4), Australia–Ireland (4), and Brazil–Portugal (3) (Figure 5).

The most utilized languages were English with 537 articles, Spanish with 16, and French with 10, followed by German (8), Italian (6), Portuguese (4), Russian (3), and Turkish (2). The most utilized categories in WOS were: vascular, heart, and thoracic surgery (n = 495), antibiotics and anti-microbials (n = 15), and nursing (n = 6).

### 3.4. Keywords

With respect to the keywords, considering the Keywords Plus, the five most utilized words were: complications (n = 107), phlebitis (n = 107), prevention (n = 80), risk (n = 64), and thrombophlebitis (n = 63).

**Figure 6:**
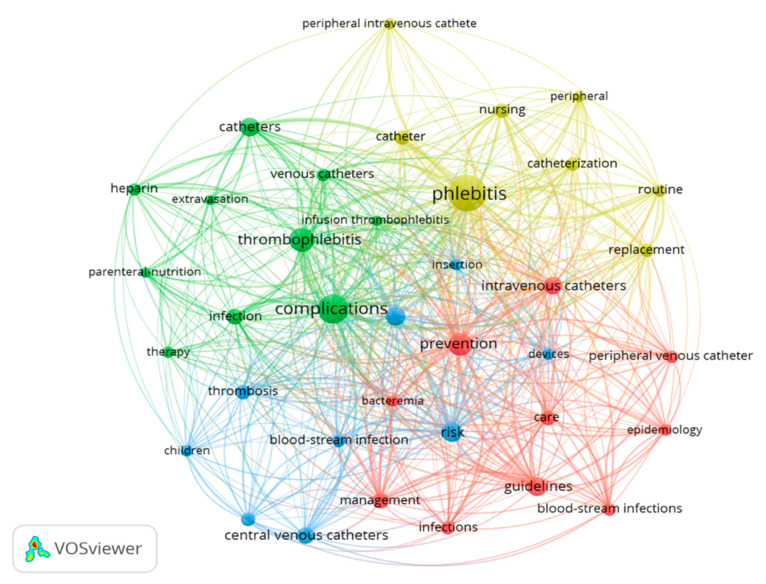
Network map of the 37 keywords with a frequency of more than 20 occurrences.

When analysing co-occurrence, the units of analysis used were all the keywords (KeyWords Plus and Author keywords) with a minimum of 20 occurrences. A total of 37 terms were identified. This strategy allows for the identification of the most relevant and significant terms in the topic being explored. In the resulting network map, it was observed that the 10 words with the greatest occurrence were phlebitis (218 times), complications (138), prevention (86), thrombophlebitis (90), and risk (65) (Figure 6).

Four clusters were differentiated in the network map:

- Cluster 1 (ten items, in red): bacteraemia, blood-stream infection, care, epidemiology, guidelines, infections, intravenous catheters, management, peripheral venous catheter, and prevention.
- Cluster 2 (ten items, in green): catheter, complications, extravasation, heparin, infection, infusion thrombophlebitis, parenteral nutrition, therapy, thrombophlebitis, and venous catheter.
- Cluster 3 (nine items, in blue): blood-stream infection, central venous catheter, children, devices, insertion, peripherally inserted central catheter (PICC), risk, risk factors, and thrombosis.
- Cluster 4 (eight items, in yellow): catheter, catheterization, nursing, peripheral, peripheral intravenous, phlebitis, replacement, and routine.

The nodes phlebitis, thrombophlebitis, complications, and prevention were interrelated. When the clusters were analysed, it was observed that all of them contained the word ‘catheter’ alone or combined with other words (intravenous catheters, peripheral venous catheter, catheter, venous catheter, central venous catheter, PICC). In addition, clusters 1 and 3 repeated the term blood-stream infection, and other complications appeared such as bacteraemia (cluster 1) and infection (cluster 2). Cluster 4 included terms related with care and nursing (catheterization, nursing, peripheral intravenous replacement, routine), while cluster 1 included words related with care and evidence-based practice (care, guidelines, management). 

Additionally, it should be mentioned that terms associated with the treatment of phlebitis due to venous access were only found in cluster 2 (heparin, therapy), with no other cluster showing words associated with the assessment of phlebitis. A more detailed analysis showed in only thirty documents (2.97%), with the word ‘treatment’ in the title, and in just thirteen documents (1.29%), the words ‘score’, ‘tool’, or ‘scale’, while the word ‘psychometric’ was only found in one article, with the latter associated with the assessment of phlebitis.

## 4. Discussion

The findings of the present study present an overall view of the scientific production on PVC-related phlebitis. With this in mind and considering the secondary aims of this study, the discussion is divided into two subsections: (1) Bibliometric elements of the scientific production; and (2) Current status and research lines in PVC-related phlebitis.

### 4.1. Bibliometric Elements of the Scientific Production

Since 1950, the year in which we found the first descriptions of phlebitis [29], the number of documents published increased exponentially until 2021, the year in which we found the peak number of contributions related to the subject (n = 51). This continuous increase in the number of publications could be due to the many different types of research related with this topic, which encompass studies on incidence, risk and related factors, prevention, care, complications, and types of catheters, among others, according to the keywords (KeyWords Plus and Author keywords) identified in this study.

As for the research areas, Nursing was found in third place (201 documents), behind areas such as General Internal Medicine (372 documents), and Cardiovascular System Cardiology (279 documents). The competence of placement of a venous catheter varied as a function of the type of catheter, but monitoring and follow-up were nursing responsibilities [30,31,32]. Different studies [30,31,32,33] corroborate the benefits obtained from continuous education based on evidence and adherence to good practices guides and protocols. Therefore, evidence-based standards such as those published in the document Infusion Therapy Standards of Practice in 2021 of the Infusion Nurses Society (INS) [34] should be a priority in their clinical use by professionals. They should be incorporated into institutional policies, procedures, and clinical practice protocols to be the basis for clinical decision-making and thus provide quality care and patient safety. These aspects would be related to cluster 1 with the terms guidelines, care, and management.

The two most cited articles refer to both centrally and peripherally inserted *catheters:* “Intravascular Complications of Central Venous Catheterization by Insertion Site” [35] with 370 citations, and “Risk Factors for infusion-related phlebitis with small peripheral venous catheter–a randomized controlled trial” [36] with 357 citations. Both belong to the area of General Internal Medicine, with the highest number of contributions classified within this category. In relation to the type of catheter and their choice of use, the INS suggests selecting the least invasive, with the smallest external diameter and the fewest number of lumens [34]. The factors that the professional must consider for the choice include: the intravenous therapy prescribed to infuse, the treatment regimen, the expected duration of the therapy in time, the characteristics of the patient (vascular, age, comorbidities, history of therapy of infusion, catheter placement preference), and lastly, the capacity and resources available to care for the device [34].

With respect to the journals, we can indicate a shared interest for the subject in specialized journals (*Journal of Intravenous Nursing, Journal of Infusion Nursing, Journal of Vascular Access*), as well as more generalist ones, most notably the *Journal of Clinical Nursing*. It is also important to highlight the relationship of this subject with the infusion of parenteral nutrition, and for this, two journals appeared (*Journal of Parenteral and Enteral Nutrition* and *Clinical Nutrition*). Cluster 2 specifically identified parenteral nutrition. Thus, there is relevant scientific production that relates peripheral parenteral nutrition with phlebitis [37,38] and, in some cases, studies were specific to children or paediatrics (cluster 3) [39].

A trend was found towards open access publications (230 documents) and the use of the English language. Although the country with the most contributions was the USA, the three authors (Rickard CM, Marsh N, and Mihala G) with the most publications on the subject resided in Australia. This can be attributed to the existence in Australia of a renowned research centre of important relevance for this topic: the Menzies Health Institute, Queensland. The two other highlighted areas are China (Asia) and Spain (Europe). China is, along with the USA, the country with the most scientific publications currently in different fields, and therefore its presence is not surprising. The results in relation to Spain can be attributed to institutional interest or productive authors in this topic and the determination to publish in English journals with an international impact. Furthermore, there is a highly relevant line of national and institutional research in Spain with specific programs such as the Phlebitis Zero Program [40], with impact at a clinical level.

Despite international interest in the subject, there is a lack of relationships between countries, as collaborative scientific production can be considered low (22 documents) given the global nature of this subject, which is a universal procedure.

### 4.2. Current Status and Research Lines in PVC-Related Phlebitis 

Phlebitis, or thrombophlebitis related with vascular access, is still an unresolved subject. Considering the word ‘epidemiology’ that appeared in cluster 1, it should be noted that the INS [34] establishes an acceptable index of incidence in clinical surroundings of 5%, although current studies [4,41] show a much higher clinical incidence, with values ranging between 31% and 55%. This enormous disparity in the scientific literature regarding the incidence of phlebitis could be explained by important differences in the studies according to definition, design, patient selection, and duration of follow-up [5]. 

Regarding complications, only extravasation or infiltration were found (cluster 2), even though they are related to phlebitis [15]. Extravasation is caused by infiltration of the treatment or intravenous infusion into the surrounding tissue. Therefore, a differential diagnosis should be made with phlebitis. In extravasation, there is local cold skin, decreased flow rate, and a difference compared to the contralateral extremity [42]. In phlebitis, the related signs and symptoms are pain, surrounding red swelling, hyperaemia, warmth, redness, tenderness, and oedema, among others [9]. However, the associated complications can lead to more severe situations, such as infection, bacteraemia, or blood-stream infection (clusters 1, 2, and 3) found in other studies [43,44].

The study by Tatsuno et al., 2019 concluded that the mortality associated with blood-stream infections in PVCs versus central venous catheters (CVCs) does not differ. Therefore, PVCs are concluded to be no safer and the same safety precautions must be used in both types, and unnecessary PVCs must be avoided.

The risk of phlebitis, as well as its prevention, is a highly studied issue and emerges in cluster 1. In short, risk can be divided into two causes: (1) Mechanical (catheter size, catheter material, insertion of cannula near articulation, method of immobilization, and dwell time); and (2) Chemical (medications with variable pH or osmolality, hypertonic solutions, bacterial causes due to contaminated solution, and patient-related aspects such as age, medical conditions, etc.) [45]. These elements generate the general and specific recommendations for the prevention of venous catheter phlebitis.

In agreement with other studies, some current reviews [9,15] show the scarcity of contributions related with the treatment of phlebitis and also highlight the lack of scientific studies. The only product that appears in this review is heparin (cluster 2). Heparin can have a double function as a treatment product and a product to maintain the patency of the catheter. In relation to the treatment of phlebitis, products can be classified into three types: (1) Physical measures (cold or heat applied topically on the skin); (2) Phytotherapeutic treatments (sesame oil, phellodendron, chamomile, calendula, aloe vera, quercetin 2%, ichthammol glycerine, alcohol compress); and (3) Pharmacological treatments (magnesium sulphate, glycerine, heparinoid) [9]. These products are mostly for topical application. Heparin and heparinoids have a topical effect intended to reduce the local symptoms associated with phlebitis. Heparin produces a percutaneous antiphlogistic and antithrombotic effect [46]. A meta-analysis carried out in 2023 [15] showed that heparinoids are the pharmacological products which most significantly reduce phlebitis and PVC infiltration, although phytotherapeutic products such as ichthammol with glycerin also achieve this effect. It should be noted that the authors state that there is little scientific evidence and low methodological quality on the most effective treatment for PVC-associated phlebitis. Additionally, another study [9] details that the physical products differ in terms of the temperature at which they should be applied and that their effectiveness is limited. The same study [9] considers that phytotherapeutic treatments have potential but are difficult to investigate since they are products which have not been approved for sanitary use in Western countries. 

In relation to its functionality as a product to ensure catheter patency, heparin use is very limited. Current studies [47] show that sodium heparin and 0.9% saline solution have the same efficacy to maintain catheter patency and prevent obstructions. It is not even possible to detail the dose, concentration, volume, and frequency of heparin to achieve this patency-maintaining catheter-sealing effect. Therefore, the evidence suggests that heparin may have little or no effect on the duration of venous catheter patency, and no differences in safety (infections, mortality, bleeding, heparin-induced thrombocytopenia) were found either [48].

This poor situation is even more evident in the assessment of phlebitis, a concept that does not appear in any cluster. Thus, more studies are needed on instruments to evaluate phlebitis and its psychometric validation [12,49,50]. Nursing practices on the management of phlebitis should be standardized and the use of a phlebitis scale should be included in this procedure [16,51].

Furthermore, regarding nursing care and replacement/routine (Cluster 4), the literature shows the complications or effects of new venipunctures such as economic cost, risk of patient stress, risk of peripheral vascular trauma, and nursing time dedicated to the procedure. Some studies do not show a direct benefit of routine substitutions every 72 or 96 h, but do rather expose the patient to the complications previously detailed. Although, some authors detail that the substitution of catheters is clinically safe.

### 4.3. Limitations

The search in WOS-WOSCC as the only data source is justified since it covers the field under study internationally. This database selection decision could imply geographic and linguistic biases of the included documents. Additionally, it should be noted that studies of this type provide information through bibliometric indicators on the quantity of scientific production but not on the quality of the studies. This could be an approach for further studies. 

## 5. Conclusions

This bibliometric analysis shows the research trends in the field of phlebitis secondary to vascular access. The main research lines are centred on investigating effective prevention strategies, risks, and associated complications. Little was found on the treatment of catheter-related phlebitis, with this study offering a very limited vision of this aspect with heparin being the only treatment subjected to research. This reflects the lack of studies and consensus in this regard. For their part, assessment tools and the integration of advanced technology for the early detection of this complication are topics that have still not been tackled in the literature. Presently, research on this subject is increasing internationally, although a more global approach is required as well as greater collaboration among researchers in different regions of the world. This would enable access to a wider dataset and the cross-validation of findings in diverse clinical environments and populations. 

## 6. Relevance to Clinical Practice

Venous access management is a nursing responsibility. Complications associated with vascular access are a problem for patient safety at the hospital level and an unresolved issue. For this reason, despite the increase in scientific production over time, it remains a topic about which more research is needed. Phlebitis is one of the most relevant complications related to venous catheters.

The present bibliometric analysis evaluates the scientific production on the topic and extracts information on: year of publication, number of references, journals, areas of interest and research, countries, languages, and keywords utilized. This information is key for researchers (novices or experts) who intend to address this topic since it allows them to quickly identify information of great interest. In addition, this analysis provides a view of the existing research landscape and serves as a phase to identify research trends, news, and innovation in this subject. 

The dimensions that should continue to be considered in new research, according to the findings of this review, are the instruments for evaluating phlebitis and their validation, and the treatment to follow in the case of established phlebitis. For this reason, studies on nursing care based on the best available scientific evidence are necessary to improve the quality of health care and for patient safety.

Finally, the present work allows us to identify key information in the literature to establish communication and collaboration between researchers, thereby facilitating scientific advancements on the prevention and management of phlebitis due to vascular access. Collaborative studies between researchers are necessary to achieve an international dimension that facilitates progress in this area. This would entail the development of scientific policies and activities, as well as dissemination of the results and their impacts [52].

## Figures and Tables

**Table 1:** Characteristics of the journals with the most relevance in different classifications.

Journal	JIF * (2021)	Edition	Research Area or Category	Quartile (2022)	JIF Rank	H-Index
Journal of Vascular Access	2.326	SCIE	Peripheral Vascular Disease	Q4	52/67	42
Journal of Infusion Nursing	0.789	SCIE	Nursing	Q3	NA	38
Journal of Clinical Nursing	4.423	SCIE	Nursing	Q1	4/125	117
Journal of Parental and Enteral Nutrition	3.896	SCIE	Nutrition and Dietetics	Q3	51/90	110
Clinical Nutrition	7.643	SCIE	Nutrition and Dietetics	Q1	13/90	161

* Journal Impact Factor.

## Data Availability

The data analysed for the current study are not publicly available due to privacy restrictions but are available from the corresponding authors on reasonable request.

## Public Involvement Statement

No public involvement in any aspect pf this research.

## Guidelines and Standards Statement

This manuscript was drafted against the “The PRISMA 2020 statement. An updated guideline for reporting systematic review” for systematic reviews research.
