# Correction: Torné-Ruiz et al. Evolution of Scientific Production on Phlebitis Secondary to Vascular Access: A 71-Year Bibliometric Analysis. *Nurs. Rep.* 2023, *13*, 1635–1647

**DOI:** 10.3390/nursrep14030164

**Published:** 2024-09-03

**Authors:** Alba Torné-Ruiz, Judith García-Expósito, Aida Bonet, Olga Masot, Judith Roca, Laia Selva-Pareja

**Affiliations:** 1 Department of Nursing and Physiotherapy, University of Lleida, 25199 Lleida, Spain; alba.torne@udl.cat (A.T.-R.); aida.bonet@udl.cat (A.B.); olga.masot@udl.cat (O.M.); laia.selva@udl.cat (L.S.-P.); 2 Hospital Fundació Althaia, Xarxa Assistencial Universitària de Manresa, 08243 Manresa, Spain; 3 Group Preving (Vitaly), 03003 Alicante, Spain; 4 Health Care Research Group (GRECS), Biomedical Research Institute of Lleida, 25198 Lleida, Spain; 5 Health Education, Nursing, Sustainability and Innovation Research Group (GREISI), 25199 Lleida, Spain

**Error in Figure**

In the original publication [1], there was a mistake in Figure 1 as published. The PRISMA figure contained erroneous data that did not match the database analysis. The corrected Figure 1 appears below. The authors state that the scientific conclusions are unaffected. This correction was approved by the Academic Editor. The original publication has also been updated.

## Figures and Tables

**Figure 1:**
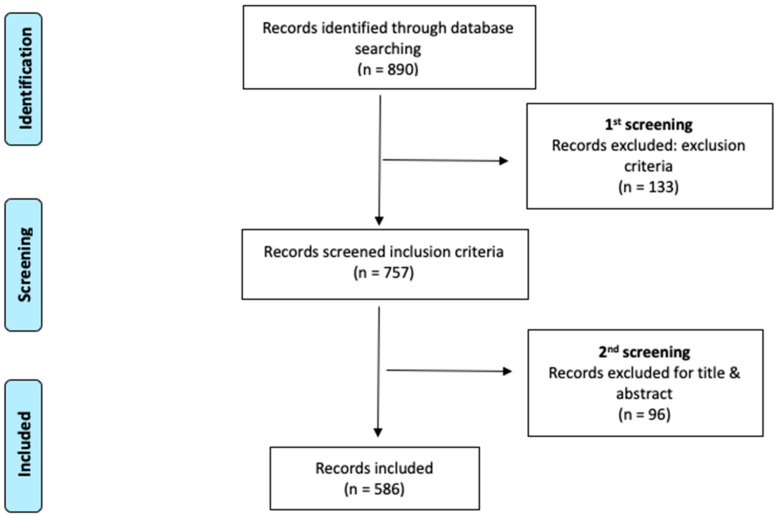
Flow diagram adapted from the Preferred Reporting Items for Systematic Reviews and Meta-Analyses (PRISMA) guidelines.
